# Advancing fabrication and properties of three-dimensional graphene–alginate scaffolds for application in neural tissue engineering

**DOI:** 10.1039/c9ra07481c

**Published:** 2019-11-12

**Authors:** Negar Mansouri, Said F. Al-Sarawi, Jagan Mazumdar, Dusan Losic

**Affiliations:** School of Electrical and Electronic Engineering, University of Adelaide Adelaide Australia said.alsarawi@adelaide.edu.au; School of Chemical Engineering and Advanced Materials, University of Adelaide Adelaide Australia dusan.losic@adelaide.edu.au; ARC Research Hub for Graphene Enabled Industry Transformation, University of Adelaide Adelaide Australia

## Abstract

Neural tissue engineering provides enormous potential for restoring and improving the function of diseased/damaged tissues and promising opportunities in regenerative medicine, stem cell technology, and drug discovery. The conventional 2D cell cultures have many limitations to provide informative and realistic neural interactions and network formation. Hence, there is a need to develop three-dimensional (3D) bioscaffolds to facilitate culturing cells with matched microenvironment for cell growth and interconnected pores for penetration and migration of cells. Herein, we report the synthesis and characterization of 3D composite bioscaffolds based on graphene-biopolymer with porous structure and improved performance for tissue engineering. A simple, eco-friendly synthetic method is introduced and optimized for synthesis of this hybrid fibrous scaffold by combining Graphene Oxide (GO) and Sodium Alginate (Na-ALG) which are specifically selected to match the mechanical strength of the central nervous system (CNS) tissue and provide porous structure for connective tissue engineering. Properties of the developed scaffold in terms of the structure, porosity, thermal stability, mechanical properties, and electrical conductivity are presented. These properties were optimised through key synthesis conditions including GO concentrations, reduction process and crosslinking time. In contrast to other studies, the presented structure maintains its stability in aqueous media and uses a bio-friendly reducing agent which enable the structure to enhance neuron cell interactions and act as nerve conduits for neurological diseases.

## Abstract 2

In this study, a bio-fabrication method has been developed for the preparation of 3D graphene–alginate composite scaffolds with great potential for neural tissue engineering.

## Graphical abstract

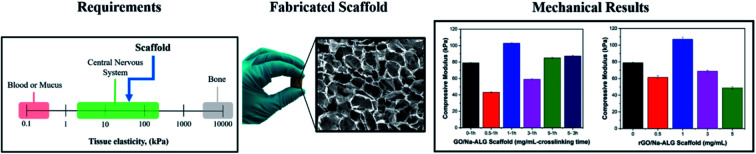

## Introduction

The most critical step in neural tissue engineering (NTE) is the assembly of bioscaffolds loaded with nerve cell sources to produce a 3D tissue substitute for transplantation.^1^ These approaches intend to develop treatments for nerve disorders and spinal cord injury (SCI) through the help of biomaterials and cell transplantation methodologies.^2^ An optimal regenerated nerve requires appropriate scaffold materials with suitable structural, mechanical, biological, and physical features that give rise to neurite and axonal growth, prevention of scar formation, and axonal alignment to the lesion area.^3^ Therefore, materials development of these scaffolds is a topical and pivotal area of research that has attracted significant research interests in recent years. Thus, there is a need for the development of new class of materials to provide enhanced features for neural tissue engineering.^4^

To address these requirements, 3D composite scaffolds have attracted great attention due to their versatile properties to overcome limitations of those prepared by a single material. The most noteworthy advantage of composite materials for tissue engineering is that their properties can be easily adapted by modifying the composition and structure of biomaterials according to characteristics of specific tissue.^5^ A number of different methods have been employed to construct porous 3D structures including solvent casting, 3D printing, gas foaming, freeze-drying, and phase separation. The freeze-drying approach is considered as one of the most common well-established methods for fabricating porous materials with controllable architecture for medical applications.^6^ This fabrication process does not involve toxic solvents and rinsing steps which is certainly safe for biomedical uses.

One of the common scaffold biomaterials with a similar structure to extracellular matrix (ECM) is alginate which is considered as a very promising natural polysaccharide polymer that can be isolated from renewable sources such as brown algae or microorganisms with applications in bioengineering.^7^ It has many advantages since it is non-toxic, biocompatible, biodegradable, relatively low-cost in comparison with other polymeric materials, easy gelation using calcium chloride and appropriate as biomaterials to help the recovery of the malfunction tissues.^8^ The properties of crosslinked alginate can be tailored by various concentrations of crosslinker as well as the crosslinking time. Although alginate-based scaffolds suffer from some limitations such as lack of mammalian cell receptors, poor ability to have control over the internal architecture, low protein adsorption capability and excessive hydrophilicity, they hold the potentials for being applied in tissue engineering due to the ease of processability and delivery of growth factors and cells.^9^ To address these limitations, alginate is combined with other materials to form biocomposites that offer biological benefits of alginate together with good mechanical strength and bioactivity of the reinforcing material.

One of the main challenges in designing a 3D composite scaffold for excitable tissues is to mimic the electrical microenvironment in order to improve cellular response and create an electrical coupling with the host tissue. As a result, conductive scaffolds have been studied extensively in the form of hydrogels, microporous materials, nanofibers, and hybrid materials to enhance the transmission of electrical signals.^10^ Among many materials, graphene and its derivatives have emerged to be extensively explored in tissue engineering, regenerative medicine and other biomedical fields owing to their exceptional electrical and mechanical properties.^11^ Particularly, graphene derivatives demonstrate unique physical, chemical, and electrical properties which can promote stem cell behaviour and enhance tissue repair. Critical characteristics of graphene-based biomaterials in NTE can be listed as their morphological structure, electrical conductivity, biocompatibility, biodegradability, and ability to induce neural differentiation.^12^ Among graphene-based materials, graphene oxide (GO) has attracted attention in tissue engineering field due to its hydrophilic nature, excellent water dispersibility, better biocompatibility, and bioactivity owing to multiple oxygen functional groups on its surface to absorb growth factors and biomolecules.^13^ Studies have shown that GO can prominently enhance the proliferation and differentiation rate of cultured neuron cells as well as bioactivity and biocompatibility of the scaffold biomaterials.^14^ Furthermore, they have demonstrated that graphene and reduced GO (rGO) are considered as permissive materials playing an effective role in astrocyte growth, neurogenesis and axonal growth in case of implantation for the nervous system disease.^15,16^

In the pioneering study by Wan *et al.*, a 3D GO–alginate composite hydrogel was prepared *via* the direct mixing method showing improved mechanical strength and Young's modulus compared to pure alginate scaffold.^17^ However, the structure suffers from poor stability in aqueous media which is a fundamental property for cell culture purposes. The addition of crosslinker in the blend solution made the structure instantly dissociated in water within several minutes which has a high probability of quick-dissolving *in vivo*. Hence, unable to guarantee good healing of tissue damage. On top of that, Sinha *et al.* recently reported the synthesis of the 3D rGO-embedded alginate scaffold by reducing GO using reducing agents such as hydrazine.^18^ However, due to the toxicity of hydrazine and its traces left in the prepared scaffold, this method is not acceptable for biomedical and tissue engineering applications.^19^ Besides, the addition of rGO in the mixing solution usually causes a poor water solubility and undesired aggregation which can adversely affect cellular behaviour and fate.^20^

To address these problems, the aim of the present study is twofold. Firstly, it attempts to improve the fabrication method of the 3D graphene–alginate composite scaffolds that uses a simple, scalable and environmentally sustainable fabrication technique involving solution mixing, freeze-drying, crosslinking, and bio-reduction. This method could enhance the stability of the 3D matrix in culture media. Secondly, it is to optimize the properties of the prepared 3D scaffold in terms of morphological, mechanical, and electrical characteristics to meet challenging requirements in neural tissue engineering. To accomplish the first aim, we developed improved synthetic procedure which enables the production of conductive scaffolds based on the *in situ* reduction of GO/Na-ALG aerogels with gelatin (a biodegradable collagen-based biomaterial) which makes the structure more favourable for cell culture. The advantage of the proposed bio-reduction process with gelatin is to eliminate the use of toxic reducing agents, which eliminated the need for using dangerous reducing agents such as hydrazine resulting in no harmful residue for neuron cells. To achieve the second goal, we explored the influence of GO concentration, reduction process, and crosslinking time of the 3D structure to establish the optimised GO content and synthetic conditions in order to achieve proper physicochemical, mechanical and electrical properties of Graphene Oxide–Sodium Alginate (GO/Na-ALG) nanocomposite scaffolds. The fabricated 3D scaffolds are characterized by a broad range of characterization methods such as Scanning Electron Microscopy (SEM), micro-CT scanning, Dynamic Mechanical Analysis (DMA), Thermogravimetric Analysis (TGA), Cyclic Voltammetry (CV), and Electrochemical Impedance Spectroscopy (EIS).

## Experimental details

In the following subsections, we describe the fabrication details of hybrid fibrous scaffolds with different concentrations of GO.

### Materials

Sodium alginate (Na-ALG) was purchased from AJAX Chemicals (Sydney, Australia). Type B gelatin powder, extracted from bovine skin was purchased from Sigma-Aldrich. Calcium chloride dried (molecular weight of 110.99 g mol^−1^, Chem-Supply) was used as the crosslinking agent. Natural graphite flakes were purchased from a local mine (Uley, Eyre Peninsula, South Australia, Australia). Potassium permanganate (KMnO_4_, Sigma-Aldrich), 98% sulphuric acid (H_2_SO_4_, Chem-Supply), 85% w/w phosphoric acid (H_3_PO_4_, Chem-Supply), and 30% hydrogen peroxide (H_2_O_2_, Chem-Supply) were used during GO synthesis. High purity Milli-Q water (18.2 MΩ cm^−1^, pH of 5.6) was used throughout the study.

### Synthesis of graphene oxide (GO)

Graphene oxide (GO) was prepared *via* the modified Hummers' method from graphite flakes.^21^ Briefly, concentrated acids including sulphuric acid (H_2_SO_4_) and phosphoric acid (H_3_PO_4_) with a ratio of 9 : 1 were mixed with 2 g of graphite and 18 g of potassium permanganate (KMnO_4_). The mixture was stirred and heated to 50 °C for 16 hours. The obtained solution was cooled with 400 mL of ice cubes and hydrogen peroxide was added to it subsequently. The brownish-colour solution was then centrifuged for two hours and washed with 30% HCl and water in order. After discarding the supernatants, the resultant GO paste was kept in the fridge for further use.

### Preparation of graphene oxide–sodium alginate (GO/Na-ALG) composite scaffold

**Fig. 1:**
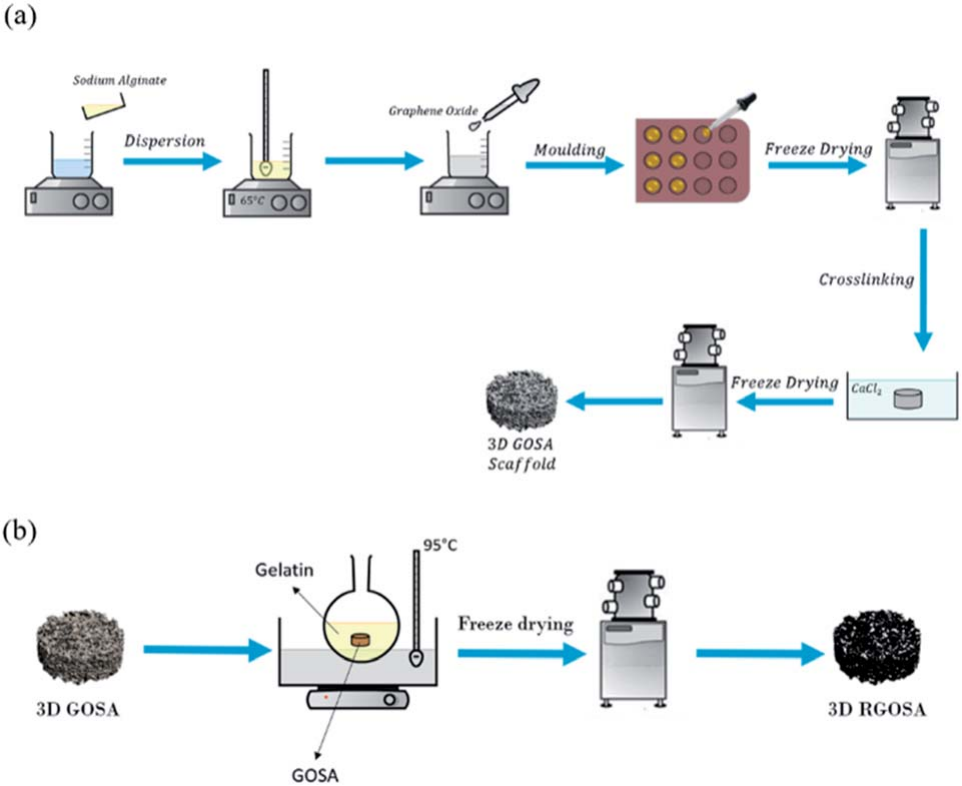
Schematic diagram of fabrication process of (a) GO/Na-ALG and (b) rGO/Na-ALG scaffolds.

The GO/Na-ALG aerogel was fabricated through incorporating GO into alginate matrix by combining freeze-drying and ionic cross-linking process, as reported earlier^22^ with slight modifications. Briefly, as summarized in Fig. 1a, the GO suspension was ultrasonic-dispersed for 15 min to obtain a homogeneous dispersion. Meanwhile, sodium alginate (4 wt%) was dissolved in deionized water and was constantly stirred to form a transparent solution. Then, GO suspension was added into the solution to get the final ratios of 0, 0.5, 1, 3 and 5 mg mL^−1^, with continuous magnetic stirring, until a homogeneous dispersion was obtained. The GO/Na-ALG mixture was poured into moulds, frozen, and freeze-dried. After that, the aerogel was immerged in 1 M CaCl_2_ solution for two different time intervals (1 h and 3 h) to achieve the calcium ion-induced cross-linking process. After cross-linked by calcium ions, the aerogel was washed with deionized water several times to remove the unbound calcium ions and was freeze-dried again to obtain the cross-linked GO/Na-ALG aerogel. The obtained GO/Na-ALG scaffolds with 0, 0.5, 1, 3 and 5 mg mL^−1^ of GO are named as Na-ALG, GO/Na-ALG0.5, GO/Na-ALG1, GO/Na-ALG3 and GO/Na-ALG5, respectively. The dimension of each sample is almost 14 × 12 mm.

### Preparation of reduced graphene oxide–sodium alginate (rGO/Na-ALG) composite scaffold

As shown in Fig. 1b, the obtained GO/Na-ALG scaffolds with various concentrations of GO including 0.5, 1, 3, and 5 mg mL^−1^ were incubated in 1 mg mL^−1^ of gelatin at 95 °C. After 24 h, the samples are washed with deionized water and sent for freeze-drying in order to get reduced GO/Na-ALG (rGO/Na-ALG) samples named as rGO/Na-ALG0.5, rGO/Na-ALG1, rGO/Na-ALG3, and rGO/Na-ALG5.

### Characterizations

The Raman analysis was done by LabRAM Evolution, Horiba Jvon (Japan) with a laser excitation wavelength of 532 nm. Fourier transform infrared spectroscopy (FTIR, Nicolet 6700) was used with a record between 4000 and 500 cm^−1^. X-ray diffraction was achieved by Rigaku MiniFlex 600 X-Ray Diffractometer (XRD) at the pipe voltage of 40 kV and the scan rate of 10° min^−1^ to confirm the oxidation of GO. The measurement was performed in the 2*θ* range from 5° to 60°. The cross-sectional area of scaffolds was cut using a blade and mounted on carbon tapes. Scanning electron microscopy (SEM) images were obtained (Hitachi, SU1510 high technologies) at an acceleration voltage of 30 kV to observe the porosity of samples. ImageJ software was utilized to analyse SEM images to measure pore sizes. The mean pore size and the standard deviations were calculated for at least 5 pores in each of the collected images (*N* = 3) per scaffold type. These pores were selected randomly for both long and short pore axes. X-ray microtomography scans were carried out using a micro-CT scanner (SkyScan 1276, Bruker micro-CT, Belgium) operated at a source voltage of 55 kV and source current of 200 μA with 0.25 mm aluminium filter. The samples were rotated to 360° with a rotation step of 0.3° and a frame averaging of two. Then, the obtained images were reconstructed by NRecon software. The volume of interest was selected in the centre of the scaffold to measure scaffold structures only. 3D images and qualitative data of scaffold formation were achieved by CTAn and CTVol softwares, respectively.

Thermogravimetric analysis (TGA, Q500 TA instruments) in a nitrogeneous atmosphere was performed to study the thermal stability of the composite structure. During this test, samples (approximately 2.3 to 5 mg) were placed in platinum crucibles and heated from room temperature to 900 °C at the rate of 10 °C min^−1^. The mechanical properties of porous scaffolds were tested with the TA Q800 DMA machine in the compression mode. The test was performed at ambient temperature with a controlled force rate of 1 N min^−1^. All samples were kept hydrated by storing them in distilled water 24 hours prior to the test. The height of the scaffolds was measured automatically at 0.1 N tare load. Wettability measurements were conducted at room temperature with Milli-Q water by the sessile drop method using a tensiometer (Attension Theta optical tensiometer). The mean contact angle was reported according to measurements of three different spots on each sample. The rheological properties of the cross-linked and uncross-linked samples before freeze-drying have been determined using an automated rheometer (MCR 301, Anton Paar) with a 49 μm cone and plate fixture.

The density and porosity of the obtained scaffolds were calculated using the following equations:^23^
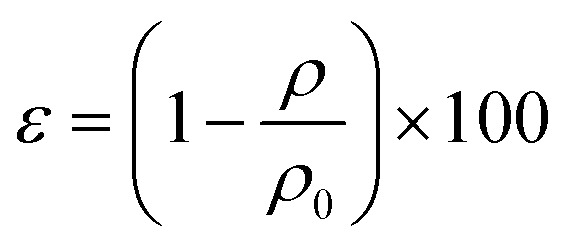
*ρ*_0_ = *ρ*_0,Na-ALG_ × *w*_Na-ALG_ + *ρ*_0,GO_ × *w*_GO_where *ε* is the percentage of porosity, *ρ* is the mass density of aerogel samples, *ρ*_0_ is the theoretical mass density, and *w* is the weight fraction of each component of the material. The densities for graphite and sodium alginate were taken as 2.2 g cm^−3^ and 1.6 g cm^−3^, respectively.

The swelling ratio of the scaffolds was determined by using the equation given below.^24^ Briefly, the dried samples were weighed and noted as *W*_0_. The scaffolds were then immersed in phosphate-buffered saline (PBS) and were incubated at 37 °C. At different time intervals, the weight of the scaffolds was measured and noted as *W*_*t*_. The experiment was carried out in triplicates.
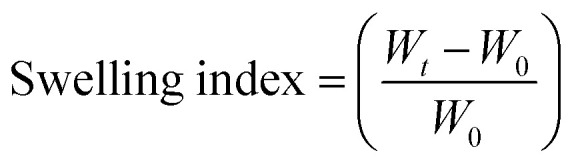


Both electrochemical measurements including cyclic voltammetry (CV) and electrochemical impedance spectroscopy (EIS) were taken using an electrochemical working station (HCH 850) with a three-electrode system in phosphate-buffered solution (PBS). CV measurements were performed at a voltage range between 0 and 1.0 V with a scan rate of 100 mV s^−1^. Regarding impedance measurements, compressed layers of conductive, rGO/Na-ALG, scaffolds were moistened in PBS and pressed between two gold electrodes. EIS was measured in the frequency range of 0.1 Hz to 0.1 MHz with a disturbance amplitude of 1 mV.

## Results and discussion

### Characterizations of GO

**Fig. 2:**
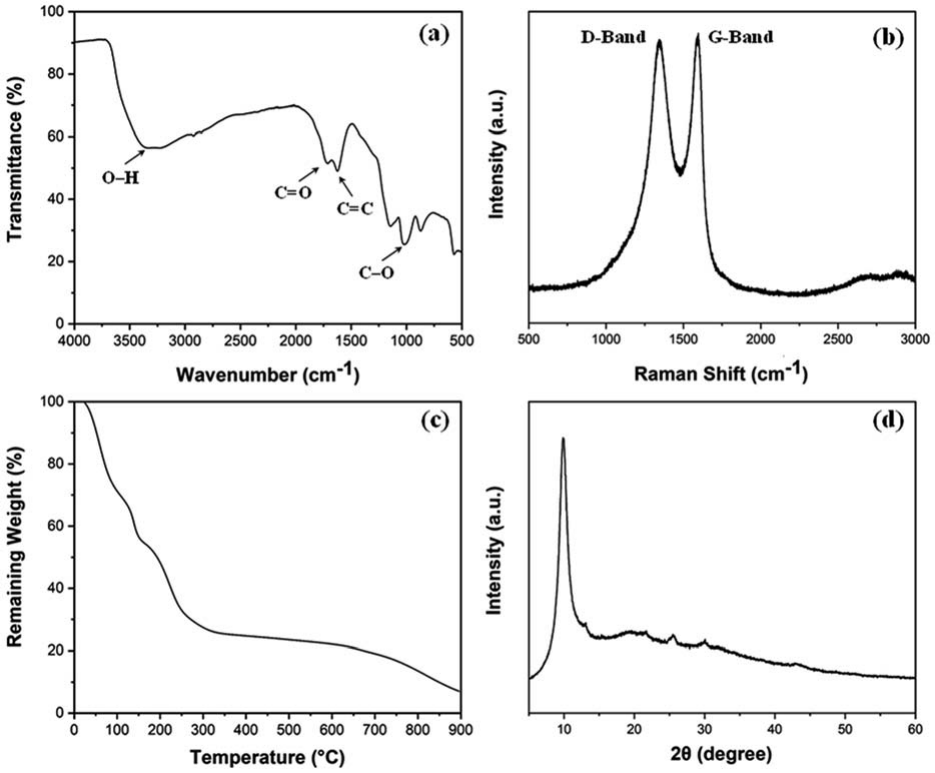
(a) FT-IR plot, (b) Raman spectra, (c) TGA curve and (d) XRD pattern of the synthesized graphene oxide (GO).

Prepared GO was characterised by a series of characterization methods to confirm their properties which are summarised below. Fig. 2a shows FTIR spectrum of GO with its corresponding functional groups. A broad peak at 3309 cm^−1^ and another peak at 1625 cm^−1^ correspond to stretching and bending vibrations of OH groups in GO. The peaks at 1706 cm^−1^ (C=O) and 1005 cm^−1^ (C–O) are caused by carbonyl, carboxylic and epoxy groups. These oxygen-containing groups on GO spectra confirm the successful oxidation of graphite.^25^

Fig. 2b shows the Raman spectrum of GO which defines its crystalline structure by considering the conjugated and carbon–carbon double bonds and structural defects. The high-intensity peaks can be observed at 1590 cm^−1^ and 1346 cm^−1^ which correspond to G-band and D-band, respectively. These typical peaks are indicative of significant structural disorders during the oxidation process. Also, the absence of the 2D (∼2600 cm^−1^) band points out that GO is dominated by the fully-disordered sp^2^ bonding network.^26^ The thermal stability of GO under nitrogen atmosphere up to 900 °C was investigated using TGA as shown in Fig. 2c. The figure highlights the weight loss around 100 °C is related to the removal of water molecules trapped inside the GO structure. The major weight loss between 200 to 400 °C can be ascribed to the thermal decomposition and complete removal of residual oxygen functional groups.^27^ The XRD measurement shown in Fig. 2d indicates the crystalline properties of prepared GO. The absence of the characteristic peak of graphite at ∼26° and the existence of the GO peak at 9.88° validates the oxidation of graphite.^28^

### Morphology and structure of fabricated graphene-based composite scaffolds

**Fig. 3:**
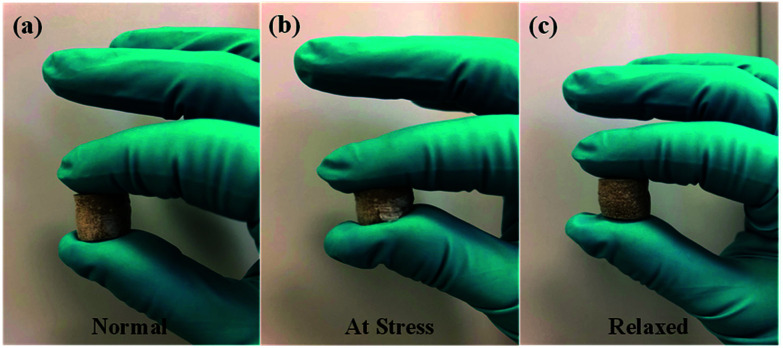
Sponge-like property of GO/Na-ALG sample in a series of events; (a) normal, (b) at stress and (c) relaxed.

Fig. 3a–c displays the sponge-like feature of fabricated graphene composite aerogels with low density. This is attributed to the optimum concentration of both GO and Na-ALG which made the composite scaffold with a proper elastic property. This sponge-like property is beneficial for the tissue engineering scaffolds to get squeezed and absorb water in culture media. So, in our experiment, the sponge was compressed a few times, in each case the structure remained almost the same with no change in structure, indicating a repeatable and reversible compressive deformability. These tests are not meant to show any stiffness properties as this has been discussed further in the mechanical properties of the Results and discussion section.

**Fig. 4:**
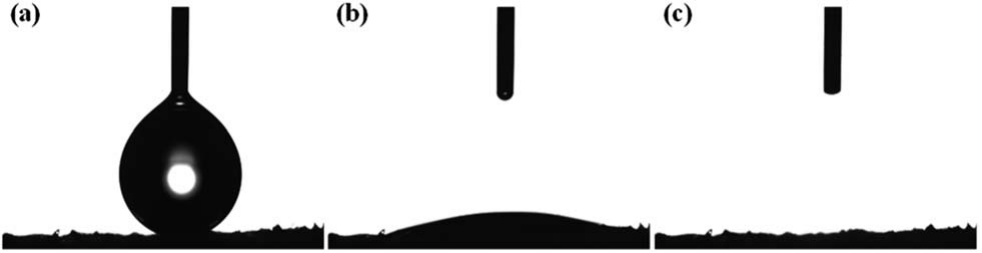
(a) Initial, (b) transition, and (c) final stage of the quick absorption and percolation of water droplet by the prepared GO/Na-ALG scaffold.

**Fig. 5:**
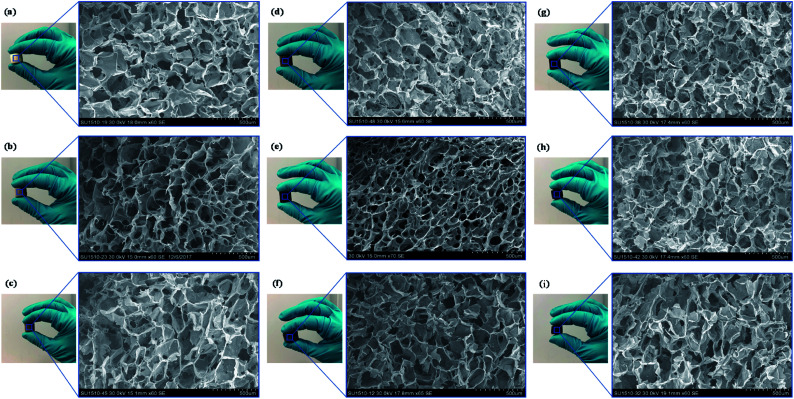
Digital photographs of fabricated graphene-based composite scaffolds with their corresponding SEM images: (a) Na-ALG, (b) GO/Na-ALG0.5, (c) GO/Na-ALG1, (d) GO/Na-ALG3, (e) GO/Na-ALG5, (f) rGO/Na-ALG0.5, (g) rGO/Na-ALG1, (h) rGO/Na-ALG3, and (i) rGO/Na-ALG5 porous scaffolds (scale bar of all images is 500 μm).

The proposed fabrication method which combines freeze-drying and crosslinking approaches suitable for tissue engineering applications improved the absorption capacities as displayed in Fig. 4a–c. The colour of GO/Na-ALG scaffolds turned to dark brown as the concentration of GO increases, as shown in Fig. 5. Besides, the black colour of reduced samples is an indication of the GO reduction. There was no significant physical difference observed in rGO/Na-ALG samples with varying concentrations of GO except changing colours. Also, the excellent stability of scaffolds has noticeably improved with physical crosslinking which is ascribed to the reaction between alginate and calcium ions to harden the scaffold and make a robust 3D interconnected-network structure.^29,30^

The microstructure of fabricated GO/Na-ALG and rGO/Na-ALG scaffolds was tested using SEM technique and summarized in Fig. 5a–j. These images revealed homogeneous 3D structures with interconnected porosity of all samples regardless of GO concentration and reduction process. The average pore size decreased from 162.5 ± 37.2 to 85 ± 16.9 μm as the GO concentration increased from 0.5 to 5 mg mL^−1^ in the GO/Na-ALG scaffolds (as tabulated in Table 1), showing the dependency of GO/Na-ALG composite on GO content. This is explained by the fact that the hydrogen bonding interaction between GO sheets and alginate resulted in increasing the resistance of forming larger ice particles in the freeze-drying method.^31^

**Table 1:** Pore diameter and porosity of the fabricated graphene-based composite scaffolds at different GO concentrations

Sample	GO (mg mL^−1^)	Pore size (μm)	Mean pore size (μm)	Porosity (%)
Na-ALG	0	132–228	162.5 ± 37.2	97.2 ± 2.3
GO/Na-ALG	0.5	112–158	147.4 ± 17.5	97.5 ± 3.5
	1	102–183	142.5 ± 28.5	98.0 ± 2.7
	3	89–150	122.6 ± 14.3	99.0 ± 3.1
	5	47–103	85.0 ± 16.9	99.3 ± 3.2
rGO/Na-ALG	0.5	93–184	116 ± 8.1	99.05 ± 3.5
	1	89–220	114.7 ± 16.1	99.18 ± 2.3
	3	82–183	98.5 ± 5.5	99.15 ± 3.4
	5	80–205	112.4 ± 24.1	99.71 ± 2.1

It should be noted that, at 5 mg mL^−1^ of GO concentration, a non-homogeneous dispersion and pore-clogging were observed and resulted in a non-spherical pore network. Unlike GO/Na-ALG composites, the average pore size remains constant for different rGO/Na-ALG scaffolds. However, a well-established porous structure as well as pore thickness were observed in the rGO/Na-ALG scaffolds after the reduction process.

Overall, in order to provide a general insight about the morphological properties of the prepared freeze-dried scaffolds comprising of alginate and graphene, the morphology of all prepared graphene-based scaffolds is regular with an average pore size of 120 ± 24 μm and high porosity ≈ 99% (±0.3%). The effective porous structure in all fabricated graphene-based scaffolds reported to be beneficial in proliferation and migration of neuron cells.^32^ It could be concluded that the porous structure has been improved by the addition of GO which has also been illustrated previously.^33^ Therefore, this facile preparation method renders the spongy graphene-based scaffold a more attractive cell culture platform due to cell penetration ability. The suggested method produces lyophilized porous scaffolds that provide stable 3D structure for cell culture and biological characterization purposes.

**Fig. 6:**
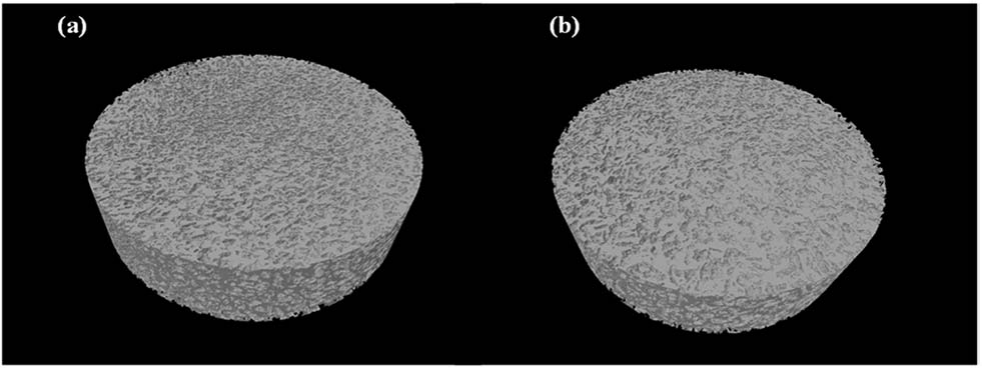
Micro-CT scan 3D models of composite (a) GO/Na-ALG0.5 and (b) rGO/Na-ALG0.5 scaffolds.

The micro-CT cross-sectional images were obtained to quantify the pore's interconnectivity and distribution of the prepared graphene-based scaffolds for cell migration and growth, as indicated in Fig. 6. Not only no skin layer was observed on both scaffolds' surface, but also uniform pores were existent from the surface through to the centre which is favourable for cellular activity by facilitating the exchange of nutrients and oxygen during tissue development. There was no significant difference observed between GO/Na-ALG and rGO/Na-ALG scaffolds in terms of μ-CT images. Therefore, the established sphere-shaped macropores allow cell colonization inside the scaffold.^34^

### Chemical composition, rheological, thermal and interfacial properties of fabricated graphene-based composite scaffolds

**Fig. 7:**
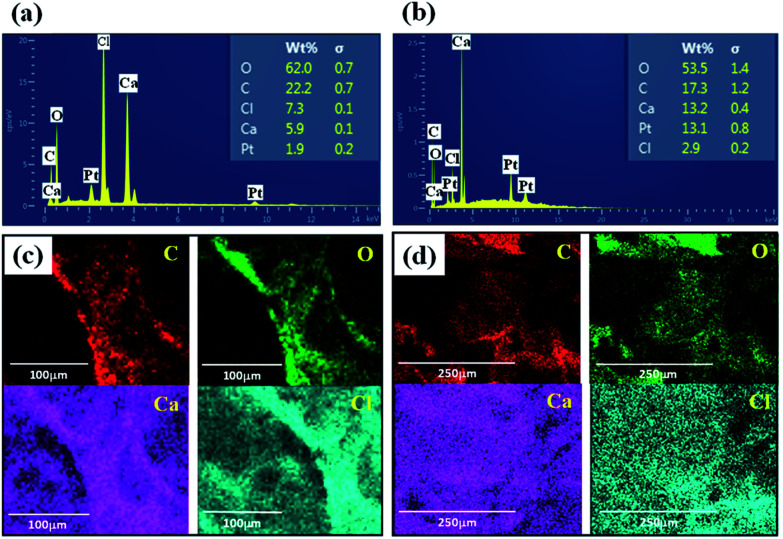
EDAX spectrum showing the elemental compositions of cross-linked (a) GO/Na-ALG and (b) rGO/Na-ALG scaffolds and mapping analysis of (c) GO/Na-ALG and (d) rGO/Na-ALG composites.

Fig. 7 presents the EDAX analysis and mapping of elements of GO/Na-ALG and rGO/Na-ALG scaffolds to have clear insights about chemical composition and homogeneous distribution of elements throughout the composite. The main elemental peaks (C, O, and Na) corresponding to graphene and Na-ALG can be undoubtedly observed from the spectra in Fig. 7a and b. Besides, calcium and chloride peaks presented in EDAX spectra indicate the diffusion of these ions in the 3D network. In addition, the elemental peaks corresponding to oxygen drops after reduction in gelatin solution, confirming the successful reduction of GO in the Na-ALG matrix.^35^

Zeta potential is another important method to estimate the performance of scaffold's material in cell culture as negative-charged surfaces are apposite for cell adhesion. The zeta potentials of prepared GO and commercial Na-ALG were about −45 mV and −17 mV, respectively. Therefore, the synthesized GO can be incorporated into Na-ALG chains in order to develop its characteristics for tissue engineering applications.^36^

**Fig. 8:**
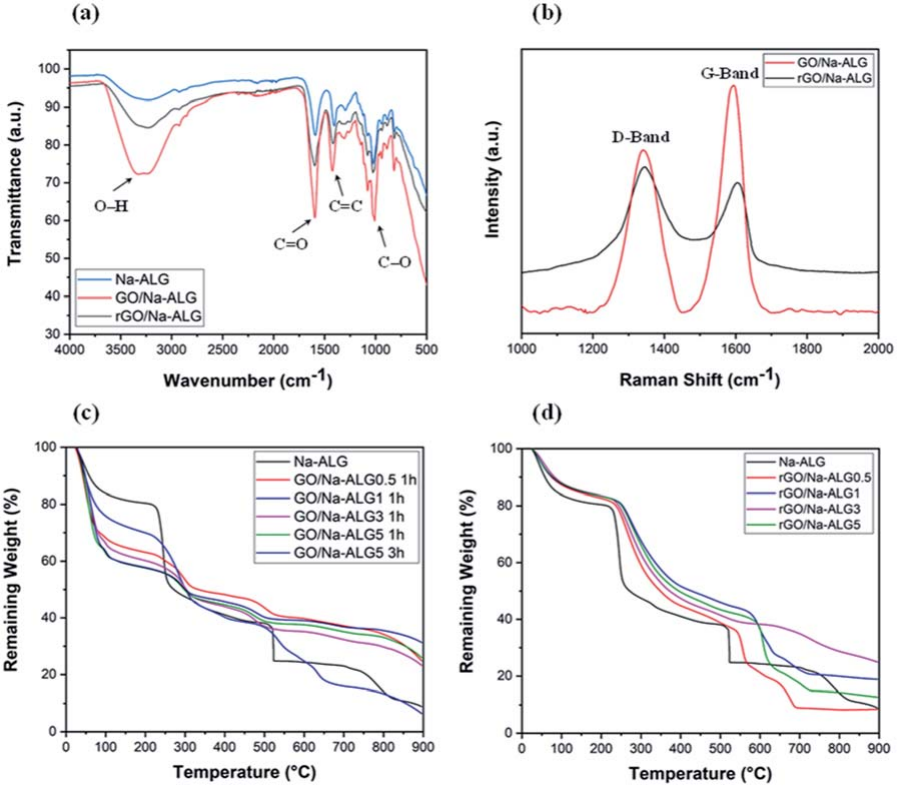
(a) FTIR spectra of composite and control samples, (b) Raman spectra of prepared composite scaffolds, TGA curves of (c) GO/Na-ALG and (d) rGO/Na-ALG scaffolds compared with control group.

FTIR analysis was specifically performed to determine the interactions between GO and Na-ALG. The spectrum basically compares the characteristic absorption peaks related to the Na-ALG, GO/Na-ALG and rGO/Na-ALG materials. As shown in Fig. 8a, the asymmetric stretching vibration of –OH and –COO– in Na-ALG caused the formation of bands at 3442 and 1597 cm^−1^. For the GO/Na-ALG sample, the peak at 1005 cm^−1^ which is attributed to C–O–C stretching is considerably stronger.

Besides, the presence of particular bands at 3316 and 1607 cm^−1^ confirms the interaction mechanism between GO and Na-ALG chains *via* intermolecular hydrogen bonds. The physical bonding between GO and alginate matrix provided a homogeneous dispersion of GO in the composite structure. Furthermore, FTIR spectra is used to study the evolution of functional groups during the self-assembly and reduction process. The most prominent and broad peak of rGO/Na-ALG was observed at 3245 cm^−1^, which corresponded to the stretching vibration of –OH bond. Meanwhile, it could be realized that Na-ALG matrix and the rGO filler had interactions through hydrogen binding as the vibration peak became broader in the composite samples. In addition, oxygen-functioning groups were less prominent after the reduction process. From the spectra, the peaks at 1593 cm^−1^, 1413 cm^−1^, and 1023 cm^−1^ which are presented in all samples can be attributed to the vibration absorption peak of the C=O, C=C and C–O bonds, respectively.^23^

Fig. 8b shows the Raman spectra of composite samples before and after the reduction process. It can be seen that both samples exhibit a prominent D-band (1340 cm^−1^) and G-band (1595 cm^−1^) in the spectrum which used to characterize carbon structure and defects. The *I*_D_/*I*_G_ intensity ratio usually points out the GO reduction. Increased value of *I*_D_/*I*_G_ from 0.71 of GO to 1.12 of rGO confirms the removal of oxygen-containing groups.^37^ The spectrum clearly confirms the reduction of the GO embedded in the composite by gelatin treatment.

Fig. 8c compares the thermal properties of GO/Na-ALG with various concentrations of GO at two different crosslinking times. Theoretically, the amount of water in the composite scaffold increases with a higher concentration of GO. It is expressed that the cause for all samples experienced the first weight loss of around 100 °C is due to the loss of absorbed moisture and trapped water. This could be due to the superior thermal conductivity of GO aiding bond cleavage.^38^ Based on analysing TGA curves, Na-ALG started to thermally degrade between 200 and 300 °C which is attributed to fracture of glycosidic bonds and removal of carboxyl and carbonyl groups of the alginate. Hence, the TGA graph confirms that GO/Na-ALG scaffolds have lower mass loss in comparison with the control group. Overall, with the addition of GO, the thermal performance of the prepared samples has improved due to hydrogen-bonding interactions between oxygen functional groups of GO and Na-ALG. As seen in Fig. 8c, samples crosslinked for a longer time period possessed higher thermal stability, which could be due to the presence of covalent bonds, as reported previously.^39^

As indicated in Fig. 8d, the curve of all the composite samples containing rGO ran above the Na-ALG aerogel since heating, which is an indication of better thermal stability of composites. All samples experienced a major mass loss at about 150 °C due to the evaporation of water molecules trapped in the nanofiber. Additionally, all scaffolds with rGO demonstrated a rapid weight loss in a similar temperature range. Overall, increasing the rGO content in the composite structure could result in a decrease in weight loss. This could be due to the better thermal stability of graphene-based materials which makes the rGO-containing scaffolds to degrade at higher temperature.^40^ It should be emphasised that the major weight loss took place at temperatures above the biological system temperature, which implies that all these structures will be very stable under human body conditions.

**Fig. 9:**
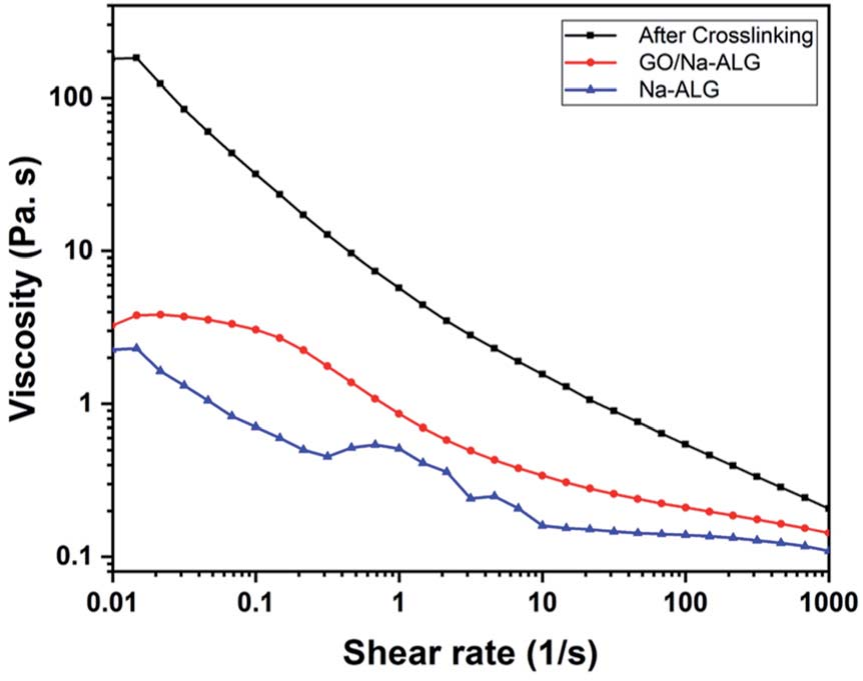
Viscosity as a function of the shear rate before/after the addition of GO and crosslinker.

The flow curves over a range of shear rates before and after the addition of GO and calcium chloride to alginate are illustrated in Fig. 9. The pure Na-ALG shows a shear-thinning behaviour. Moreover, the addition of GO apparently enhanced the viscosity of the control sample. After crosslinking, it can be admitted that the viscosity of the cross-linked solution significantly improved by two orders as a result of a strong physical crosslinking between Na-ALG and calcium chloride, which was also reported previously.^41^ It should be highlighted that immersion of various samples in the crosslinking solution could not be further analysed in terms of rheological properties. Instead, a comprehensive analysis of samples regarding mechanical properties had to be performed by means of a mechanical test machine which will be covered in the following section.

**Fig. 10:**
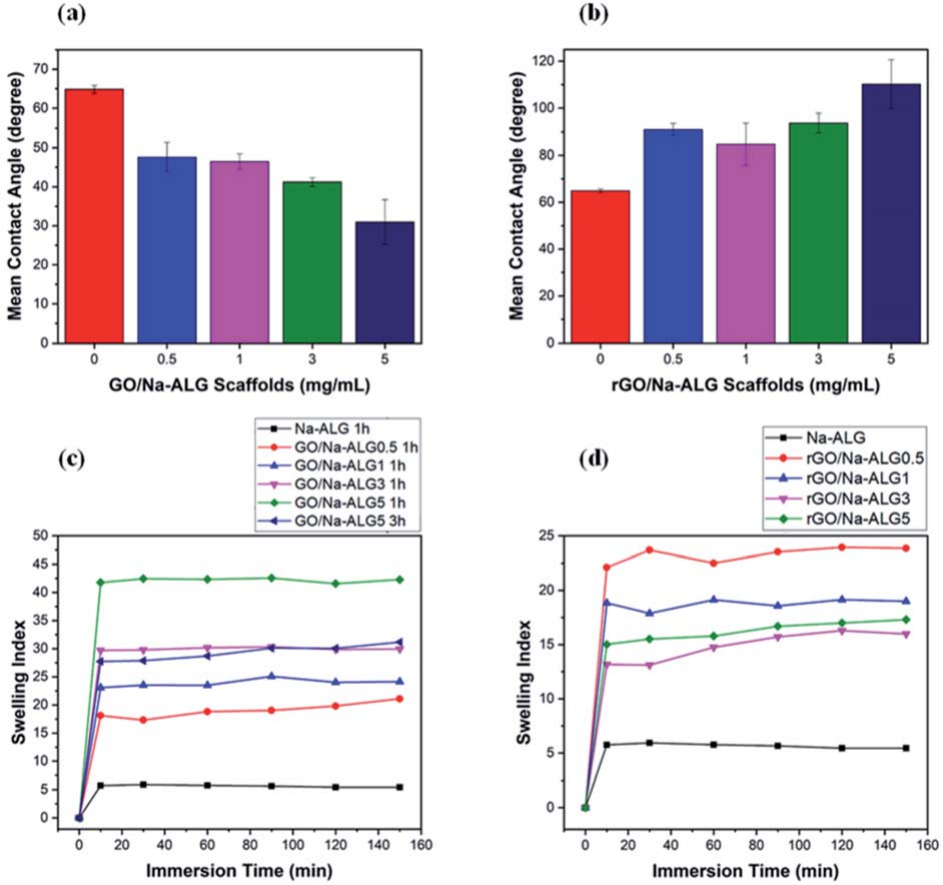
Water contact angle measurements of (a) GO and (b) rGO-based scaffolds, swelling behaviour of (c) GO/Na-ALG and (d) rGO/Na-ALG scaffolds against time.

As previously reported,^40^ an appropriate hydrophilic surface of the scaffold could give rise to the improvement of cellular affinity and adhesion. The apparent water contact angle on prepared scaffolds with different contents of GO is given in Fig. 10a and b. According to Fig. 10a, the water contact angle on the surface of composite GO/Na-ALG scaffolds decreases with the increase of GO concentration, which corresponds to the hydroxyl groups and carboxylic groups on the GO surface. For instance, the contact angle reached its minimum value of 30.9 ± 5.7° in GO/Na-ALG5 scaffold which has the highest GO content. As a result, the control samples with no GO have been improved in terms of surface morphology and wettability for better cell adhesion and proliferation. Thus, in agreement with previous works, the addition of GO could enable the interactions with biological molecules owing to its proper hydrophilicity.^42^ In the case of rGO/Na-ALG scaffolds, as indicated in Fig. 10b, reduction of samples impacted the wettability of the composite scaffolds where the hydrophilicity decreases with the increment of rGO concentration (from 91.07 ± 2.6° to 110.24 ± 18.4°). This effect could be attributed to the surface nanostructure of rGO layers.^10^ Hence, the hydrophilicity of the prepared rGO-based composite is still in a safe range for cell culture purposes. Comparing the hydrophilicity of scaffolds before and after reduction, it can be seen that scaffolds had a significant increase in contact angle after reduction of GO due to the removal of some oxygenated groups during the reduction process.

As an efficient biomaterial in tissue engineering, once the scaffolds are placed in culture media, they should swell and maintain the body fluid in their 3D network. Fig. 10c and d indicate the water absorption capability of 3D GO/Na-ALG and rGO/Na-ALG scaffolds at 37 °C at various time intervals. In the case of GO/Na-ALG scaffolds, incorporation of GO considerably increase the water uptake ability of Na-ALG from 5 up to 42 when mixed with 5 mg mL^−1^ of GO. This could be due to the hydrophilicity of GO nanosheets. On the other hand, reduction of GO/Na-ALG caused the swelling ratio of rGO/Na-ALG scaffolds to decline when GO concentration increases, which is ascribed to removing some of the oxygen-containing groups during gelatin and thermal treatment.

The swelling index of the rGO composites found to be varied from 15 to 44, depending on the GO content. Also, crosslinking time is inversely associated with a swelling ratio of fabricated scaffolds, as expected. Moreover, all fabricated composite scaffolds could clearly swell to equilibrium without dissolving, which is a critical feature for cell culture purposes. To sum up, the swelling behaviour of the prepared scaffolds could be controlled by the incorporation of graphene and reduction process.

### Electrical properties

**Fig. 11:**
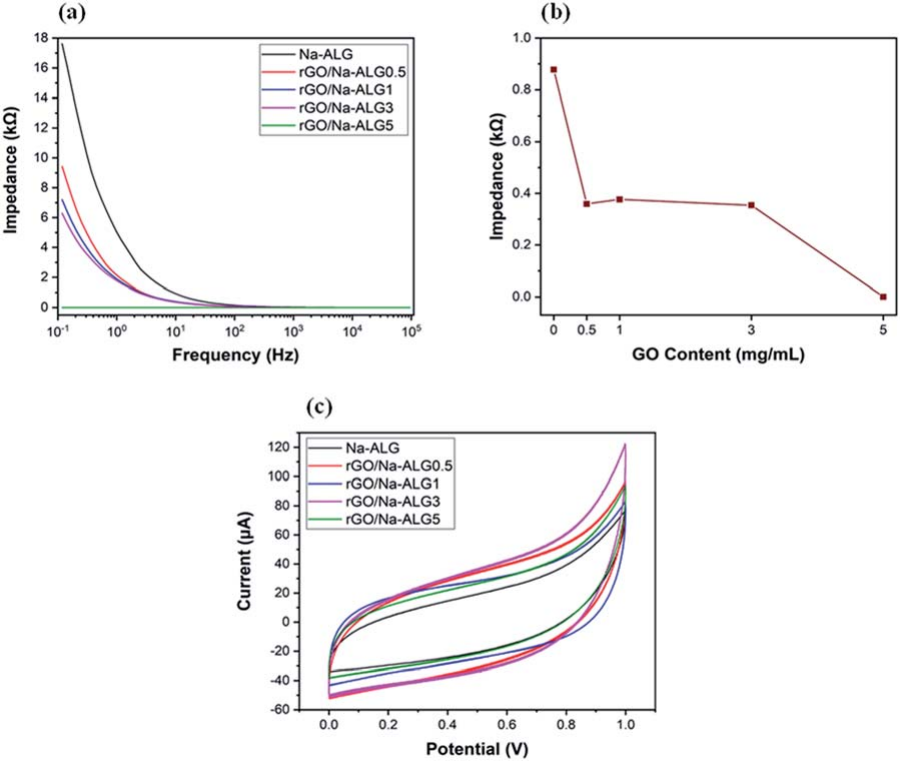
(a) A comparison of the impedance magnitudes of conductive rGO/Na-ALG scaffolds, (b) impedance *vs.* GO concentration of scaffolds at the frequency of 10 Hz, and (c) the CV curves obtained for rGO-based scaffolds containing 0 to 5 mg mL^−1^ of GO.

The characterization for electrical properties presented in a recent study by Shin *et al.* is adopted in the following electrical impedance measurement.^43^Fig. 11a, in which the EIS curve is illustrated, compares the conductivity of prepared samples over a frequency range. The control sample containing Na-ALG showed the highest electrical impedance value over the frequency range with a maximum value of ∼18 kΩ. Meanwhile, the addition of 0.5 mg mL^−1^ of GO could significantly decrease the impedance by half. Also, there is a significant drop in impedance value from 17.6 kΩ of pure Na-ALG to 6.2 kΩ of rGO/Na-ALG3. The impedance measurements were noticeably lower for samples after the reduction process due to the excellent conductivity of graphene sheets. Moreover, as the concentration of GO increased in the composite scaffolds, the conductivity is further increased which is expected and consistent with previous studies.^43^ However, there is a certain limit for the addition of GO sheets depending on their dispersion in alginate matrix to develop the electrical conductivity. This fact has been similarly revealed in previous research works, stating that there exists a crucial concentration of GO, known as percolation threshold, which is a deciding factor in maintaining the balance between GO nanosheets and GO-matrix interactions to improve the electrical conductivity.^44^

In order to explore the effect of GO concentration on electrochemical properties of scaffolds, the impedance values were plotted at a constant frequency of 10 Hz and presented in Fig. 11b. This figure illustrates that the increment of GO content from 0.5 to 3 mg mL^−1^ in the nanocomposite scaffolds led to a major increase in conductivity, which could be helpful in signal propagation and electrical coupling between neural cells and the scaffold within an injured nerve tissue. On the other hand, the addition of more GO content abruptly decreased the impedance value to 3.14 × 10^−4^ kΩ for GO/Na-ALG5 scaffold. At this condition, the agglomeration and formation of resistant path in the structure can probably result in an overestimated impedance value.^45^

The electrochemical properties of fabricated conductive scaffolds in PBS buffer are indicated in Fig. 11c by cyclic voltammetry curves. It can be seen that the current is delivered mainly through charging/discharging the interfacial double layer. Additionally, the current densities associated with the rGO scaffolds are evidently higher than that of the control scaffold which is indicative of higher double-layer capacitance due to a larger specific surface area. Therefore, the incorporation of graphene in the matrix could cause a stronger charge injection ability which is an effectual factor for neural stimulation.^46^

### Mechanical properties

**Fig. 12:**
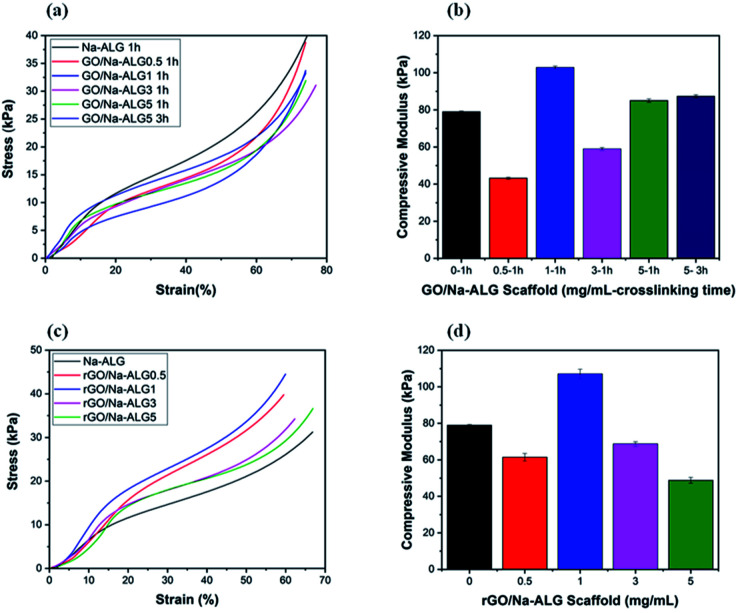
Stress–strain curves of (a) GO/Na-ALG and (c) rGO/Na-ALG scaffolds, and the corresponding compressive modulus of (b) GO/Na-ALG and (d) rGO/Na-ALG scaffolds prepared from different blending ratio.

From the mechanical point of view, every scaffold in tissue engineering must have proper mechanical characteristics, according to biomechanical properties of the targeted tissue, not only to support tissue regeneration but also, to maintain satisfactory integrity at the site of implantation during cell growth. The mechanical properties of the samples were investigated to firstly study the influence of the reduction process and different biomaterials used on the compressive modulus of the microfabricated scaffolds and secondly assess the exact match with the mechanical strength of spinal cord. The typical compressive stress–strain curves of the prepared scaffolds with different GO weight ratios are displayed in Fig. 12a and c and the compressive modulus was determined as the slope of the linear region of the curve. From the stress–strain curves, it can be observed that the stress of all prepared scaffolds has a sharp increase which is due to the solidification of hydrated porous samples exposed to a strong compressive force.^47^ Thus, the calculated compressive modulus values at 10% strain before the porous structure is damaged are plotted in Fig. 12b and d. It can be concluded that the addition of GO and increased crosslinking time enhances the mechanical performance of the scaffold. It is worth noting that, the compressive modulus of scaffold crosslinked for 3 h (0.873 kPa) had a negligible improvement compared to that of crosslinked for 1 h (0.850 kPa). The composites containing 1 mg mL^−1^ of GO, both before and after reduction, showed the highest mechanical improvement compared to other samples. This result is consistent with other studies utilizing graphene-based nanocomposites with optimum GO content of 1 mg mL^−1^.^48^

Also, the measurements confirmed that compressive strength decline to increase for GO at higher concentrations because of agglomeration. This deterioration of compressive modulus could be attributed to the defects or aggregation of GO during the fabrication procedure. This has been similarly reported in a recent study by Abzan *et al.*, which concluded that enhanced elastic modulus of graphene-based scaffolds is greatly depended on dispersion of GO nanosheets in the composite matrix.^49^

Our results reveal that the mechanical characteristics of GO/Na-ALG and rGO/Na-ALG scaffolds can be adjusted by controlling the GO concentration or graphene/alginate ratio. In addition, the nervous system tissue has a unique mechanical characteristic that meaningfully affects neural cell behaviour and tissue regeneration. It has been reported that the elastic modulus of the spinal cord varies from approximately 3–300 kPa.^50,51^ This indicates that the mechanical property of fabricated scaffolds matches up with the *native tissue* which is needed to support neuron growth. Although the mechanical stiffness of high molecular weight pure Na-ALG-based porous scaffold is adequate to match the neuron microenvironment, the incorporation of GO has brought excellent biological, chemical and physical features to the composite scaffold while keeping the elasticity in the proper range for neural tissue engineering. Furthermore, the scaffold strength could be significantly reduced due to the hydrated condition of measurements as a large amount of water deteriorates the structure's resistance to external force.^52^

Overall, aggregation was observed in higher concentrations of GO, more than 3 mg mL^−1^, for GO/Na-ALG and rGO/Na-ALG composites in which the graphene sheets were not able to fully exfoliate in the matrix. It has been observed from previous studies that the extent of improvement upon addition of GO mainly depends on the level of dispersion of graphene sheets in the matrix. Thus, the concentration of GO in the plating solution can play a significant role in generating homogeneous distribution of graphene sheets in the composite. Also, a certain concentration of GO leads to a stable dispersion owing to the van der Waals interaction of monolayers. On the other hand, an excessive amount of GO could undesirably affect the composite properties because of graphene sheets agglomeration causes a dramatic reduction in the optimal ratio of the reinforcement. A proper concentration of GO in the composite material is expected to decrease the tendency of flakes to restack and develop the permeability.^61,62^

In general, interconnected porous structure, matched mechanical strength with neural tissue and electrical conductivity of bioscaffolds make them more suitable for neural tissue engineering applications.^63^Table 2 summarizes properties of the most relevant composite scaffolds in neural tissue engineering as well as the graphene-based hybrid scaffold presented in this study. There are a number of studies that uses conductive polymers for the fabrication of neural constructs. However, poor interaction with cells and biodegradability are some of the key challenges associated with using conductive polymer-based scaffolds.^64^ In addition, the mechanical strength of the fabricated scaffold should match the nervous system tissue that is going to be engineered. This can greatly impact the cell fate, nerve regeneration, and transplantation outcome. Therefore, the prepared graphene-based scaffold reported here, which can be rendered to a conductive platform for electrical stimulation purposes, with integrated porous structure and matched mechanical strength to the spinal cord opens possibilities for further biological experiments.

**Table 2:** Comparing the previous relevant composite scaffolds for neural tissue engineering with the fabricated scaffolds in the current study

Material	Interconnected porous structure	Conductive	Matched mechanical properties with the targeted tissue	Ref.
Chitosan/gelatin/hyaluronic acid/heparan sulfate	✓	✗	N/A	53
Collagen/chondroitin sulphate	✓	✗	N/A	54
Polypyrrole (PPy) doped by butane sulfonic acid	✗	✓	N/A	55
PVV/PANi	✗	✓	✓	56
PLGA/PEG	✓	✗	N/A	57
PANi/PEGDA	✓	✓	N/A	58
PPY and PDLLA	✗	✓	N/A	59
PCL/PANI and PLGA/PANI	✓	✓	N/A	60
GO/Na-ALG and rGO/Na-ALG	✓	✓	✓	Current study

## Conclusions

In this paper, the fabrication and characterization of composite sponge scaffolds composed of Na-ALG and GO are described. The fabrication process uses solution mixing, freeze-drying and crosslinking which results in an improved, simple, eco-friendly, high yield strength and integrity 3D scaffold structure with desirable pore sizes greater than 50 μm needed for neuron cell culture. The experimental results showed that although the composite scaffold has water uptake ability, the integral composite scaffold can float on the liquid surface. Results showed that prepared scaffold properties depend on several key parameters such as scaffold materials, concentration of composite ratio, crosslinking condition, and GO concentration which can be tuned to achieve the best characteristics needed from an application point of view. In addition, the incorporation of GO improved the hydrophilicity of 3D composite scaffolds in which the water contact angle was decreased from 64.8° to 30.9° when the concentration of GO was increased from 0 to 5 mg mL^−1^, respectively.

Furthermore, electrical measurements have shown that when GO content in the composite structure increased, the conductivity of the prepared rGO/Na-ALG scaffolds significantly increased during the reduction process, which can be helpful in signal propagation for electrical stimulation purposes. Also, the compressive modulus of the scaffolds has improved to more than 100 kPa after the addition of 1 mg mL^−1^ of GO. This reported value of compressive modulus is in the reported range of mechanical strength of human neural tissue. Further, the crosslinking time can be used to tune the mechanical strength and water adsorption capacity of scaffolds according to the engineered tissue requirements. Overall, the introduction of graphene, apart from physical, electrical and chemical properties improvements, provides favourable properties for neural tissue engineering. These results suggest that the obtained porous scaffold could serve as a suitable matrix to support cellular responses for the three-dimensional culture of neural cell types. Hence, the future direction can be directed toward further biological investigations to confirm the effective capability of the fabricated scaffolds in neural induction.

## Conflicts of interest

There are no conflicts to declare.

## Supplementary Material
